# Structural, Thermal and Magnetic Analysis of Two Fe-X-B (X = Nb, NiZr) Nanocrystalline Alloy

**DOI:** 10.3390/ma16010155

**Published:** 2022-12-24

**Authors:** Kaouther Zaara, Jason Daza, Wael Ben Mbarek, Joan-Josep Suñol

**Affiliations:** Department of Physics, Campus Montilivi s/n, University of Girona, 17003 Girona, Spain

**Keywords:** mechanical alloying, nanocrystalline, structural analysis, magnetic analysis, thermal analysis

## Abstract

High-energy ball milling was used to produce two Fe-X-B (X = Nb, NiZr) nanocrystalline alloys. X-ray diffraction (XRD), differential scanning calorimetry (DSC), and vibrating sample magnetometry (VSM) were used to analyze the microstructure, thermal, and magnetic characteristics of the milled powders, the agglomerated particles (also generated during the milling process), and the compacted specimens of both alloys. The main crystallographic phase is always a bcc Fe-rich solid solution; whereas a minor Nb(B) phase is detected on powders and agglomerated particles in the Fe_80_Nb_8_B_12_ alloy. The crystalline size of the Fe_80_(NiZr)_8_B_12_ alloy is between 11 and 14 nm, whereas in the Fe_80_Nb_8_B_12_ alloy, it ranges between 8 and 12 nm. Microstrain and dislocation density are higher in agglomerated samples for both alloys than in milled powders. Thermal analysis detects structural relaxation and crystal growth exothermic processes with high dispersion in the temperature intervals and in the calculated apparent activation energy of the main crystallization process. Regarding magnetic behavior, the coercivity values of all powdered-agglomerated specimens were around 800 A/m. The coercivity is higher in compacted sample, but controlled annealing favors enhanced soft behavior.

## 1. Introduction

Fe-based nanocrystalline alloys have received widespread research because of their higher magnetic properties and exceptional physical characteristics, such as low coercivity, high saturation magnetic flux density and effective permeability with respect to those exhibited by conventional microstructures [1,2]. Nanocrystalline Fe-M-B alloys, also known as Nanoperm-type alloys where M is an early transition metal, have attracted attention due to their remarkable soft magnetic characteristics. These alloys are employed as ultrasoft magnets in a variety of commercial applications, including telecommunications, microdevices, and power electronics [3]. The above alloys are often created as amorphous using rapid solidification processes; however, a nanocrystalline structure was detected following further annealing [4]. Another production method to directly manufacture nanocrystalline alloys with these compositions is mechanical alloying (MA) of elemental powders. Mechanical alloying has developed into a very powerful method to directly create metastable microstructures, such as nanocrystalline microstructures, supersaturated solid solutions, amorphous structures, etc. [5,6]. It has been discovered in the mechanically alloyed Fe_94-x_Nb_6_B_x_ alloys (x = 9, 14, 20), the Fe lattice parameter increases with higher B doping, whereas crystallite size is reduced [7]. In the mechanically alloyed Fe_62_Nb_8_B_30_ powders, a combination of α-Fe, Nb(B), and highly disordered Fe (Nb, B) solid solution has been formed after 25 h, whereas the paramagnetic amorphous structure is attained after a longer milling time [8]. Mechanical alloying has been used to investigate the Fe_75_Nb_10_B_15_ and Fe_85_Nb_5_B_10_ systems. After milling, only Fe_75_Nb_10_B_15_ alloy produces an amorphous phase, whereas Fe_85_Nb_5_B_10_ alloy produces a bcc supersaturated solid solution [9]. Alleg et al. analyzed the magnetic and structural characteristics of mechanically alloyed Fe_75_Si_15_B_10_ powders. They observed that dispersing B and Si into the Fe lattice results in the creation of bcc-Fe(Si,B) solid solution, with coercivity and magnetization values of 55 Oe and 6.7 emu/g, respectively, after 150 h of milling [10]. Chabi et al. investigated the influence of boron content on the microstructural, structural, and magnetic characteristics of Fe_92-x_Nb_8_B_x_ (x = 5, 10, 15, and 20) alloys and discovered FeB boride is seen for higher boron amounts (x = 15 and 20). According to their results, when the B concentration increases, the amorphous relative proportion and coercivity increase, while saturation magnetization decreases [11]. In another study by Mnasri et al., Fe_71_Nb_23_B_6_ nanocrystalline alloy is produced by milling. They reported the main phase in both alloys is always bcc Fe-rich solid solution. Nonetheless, following milling, a small Nb(B) phase is found, and a minor amorphous phase is formed after 200 h of milling [12]. Likewise, Ipus et al. investigated the magnetic characteristics of FeNbB milled alloys with crystalline and amorphous boron. Their findings revealed amorphous boron was more effectively integrated into the matrix than crystalline boron, especially for short milling durations [13]. It is evident the preparation conditions affect the final product and the mechanically alloyed powders’ properties. The mechanical alloying technique is characterized by the repeated welding and fracturing of powder particles in a high-energy ball mill, which frequently results in excessive cold welding and ductile particle aggregation [14]. Even though agglomeration is a significant issue in the preparation process when the particulate size is reduced to the micron level, Hong and Kao [15] successfully incorporated very fine SiC particulates with a mean particle size of 0.3 mm into an Al matrix using the mechanical alloying (MA) process, which was used to improve the agglomeration of the reinforcement particulates as well as to reduce their crystalline size. This method is basically a ball-milling process in which the powder particles are exposed to high-energy impact [16]. Since the presence of agglomerated particles was detected throughout the mechanical process in our study, a detailed evaluation of the agglomerated particles is required. As a result, the current work aims to investigate the morphology, structural, microstructural, thermal, and magnetic characteristics of the Fe_80_-X_8_-B_12_ (X = Nb, NiZr) (at. %) alloy powders and the agglomerated particles formed by mechanical alloying for 40 and 80 h of milling. Nanocrystalline powders (80 h of milling) were compacted. Microstructure, thermal stability, and magnetic response were investigated before and after compaction. Finally, annealing was performed to improve the soft magnetic properties of the compacted specimens.

## 2. Materials and Methods

The mechanical alloying process was conducted in a high-energy planetary ball mill (Fritsch Pulverisette P7). The milling started with pure element and compound powders, Fe with a purity of 99.7% and a particle size of less than 8 μm; Nb with a purity of 99.85% and a particle size of less than 74 μm; B with a purity of 99.6% and a particle size of less than 50 μm; and prealloyed Ni_70_Zr_30_ powders (purity of 99.9% and particle size greater than 150 μm). Fe_80_(NiZr)_8_B_12_ and Fe_80_Nb_8_B_12_ were the nominal compositions produced and evaluated. Milling was conducted during 40 and 80 h under an argon atmosphere with a ball to powder mass ratio of 5:1. To analyze the agglomerated particles formed after 40 and 80 h for each alloy, samples were separated and crushed into powders using a mortar and pestle. Likewise, the final powders (80 h of milling) were consolidated to obtain bulk specimens. In addition, taking into account thermal analysis data, annealing at selected temperatures was performed. Six samples of each composition were prepared (two before annealing, two after annealing at 300 °C, and two after annealing at 600 °C). Measurements were performed in open magnetic circuit by applying OIEC 60404-7 method B (to measure test specimens almost geometry independent).

Scanning electron microscopy (SEM) with a Zeiss DSM 960A instrument was used to examine the morphology of the MA powders and agglomerated particles (Zeiss, Oberkochen, Germany). The compositions of the specimens were checked by inductive coupled plasma (ICP) in a Liberty-RL ICP Varian device. The powders, agglomerated particles, and compacted specimens were microstructurally characterized by x-ray diffraction (XRD) in a Bruker APEX D8 Advance diffractometer (Bruker, Billerica, MA, USA) using Cu-K radiation at a wavelength of λ = 1.5406 Å. The Rietveld refinement approach was used to refine the diffraction patterns by employing MAUD software (version 2.8). Thermal characterization was carried out using a Mettler-Toledo DSC30 device utilizing differential scanning calorimetry (DSC) under argon atmosphere in a temperature range from 100 °C to 600 °C at heating rates of 5, 10, 20, 30, and 40 °C min^−1^. All samples were magnetically characterized using magnetometry at room temperature with magnetic hysteresis loops in a SQUID MPMS-XL apparatus (Quantum Design; San Diego, CA, USA). The specimens analyzed and its labels are given in the Table 1.

## 3. Results and Discussion

### 3.1. Morphology Analysis

**Figure 1:**
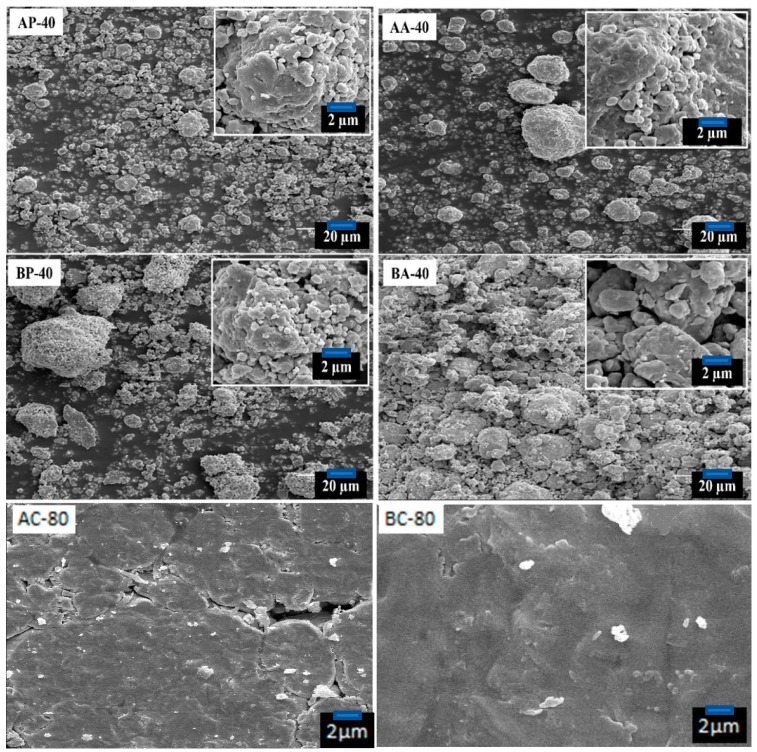
SEM images corresponding to samples AP-40, AA-40, BP-40, BA-40, AC-80, and BC-80.

The morphologies (SEM micrographs) for samples AP-40, AA-40, AC-80, BP-40, BA-40, and BC-80, are presented in Figure 1. A noticeable difference in the uniformity of the particle size (micrometric range) for powders and agglomerated particles of both alloys can be observed. The comparison was possible in sample A (Fe_80_(NiZr)_8_B_12_) because the agglomerate particles deagglomerated easily, allowing the particle size distribution to be evaluated. The formation of agglomerates is caused by particle deformation and welding during milling.

**Figure 2:**
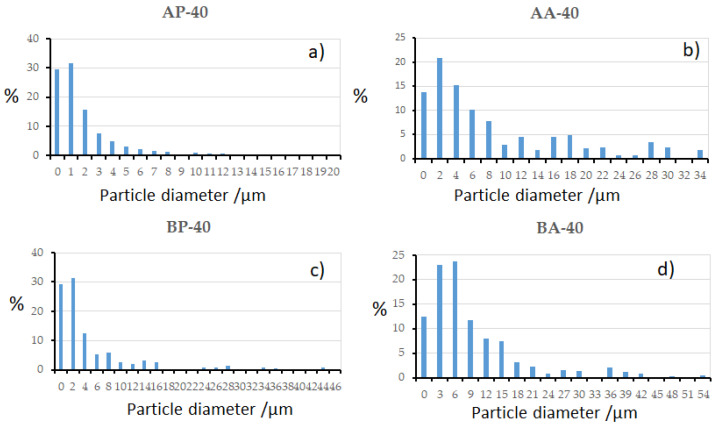
Particle size distribution of: (**a**) AP-40, (**b**) AA-40, (**c**) BP-40, and (**d**) BA-40.

The particle size distributions milling are given in Figure 2. First, for the AP-40, the majority of the particles are found below 3μm with diameters of up to 10 μm. On the other hand, as predicted, there is a significant increase in particle diameter between the powder and agglomerate forms. For AA-40, the majority of the particles are found below 10 μm with diameters of up to 34 μm. Second, for B (Fe_80_Nb_8_B_12_) powders milled for 40h (BP-40), the vast majority of particles are less than 4 μm. However, due to the sample’s tendency for agglomeration, we find diameters of up to 46 m in this case. For the same alloy in the agglomerated phase (BA-40), as expected and clearly visible on the SEM image, the majority of the particles are found below 15 μm with diameters of up to 55 μm. The agglomeration tendency is explained by the fact the Nb in this alloy has a low density, high ductility, and good formability in comparison to other elements [17]. Regarding the detected asymmetry of the distributions, the kurtosis values are 2.716, 1.501, 4.314, and 1.247 for alloys AP-40, AA-40, BP-40, and BA-40, respectively.

Regarding the composition checked by ICP, the results show the composition ratios of Fe, Ni, Zr, Nb, and B are similar in powders and agglomerates to the nominal composition (and also in bulk specimens).

Lastly, as seen in the SEM micrographs of the compacted samples, the BC-80 exhibited a higher tendency to compact than the AC-80, which showed visible holes and pores.

### 3.2. XRD Analysis

**Figure 3:**
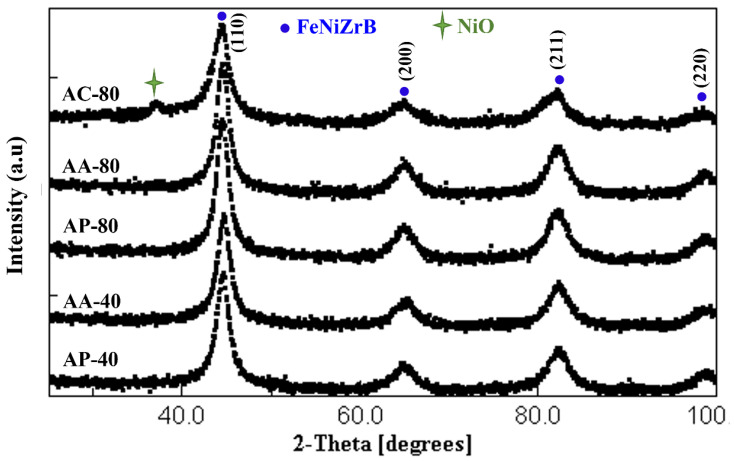
XRD diffractograms for samples of A alloy (Fe_80_(NiZr)_8_B_12_).

**Figure 4:**
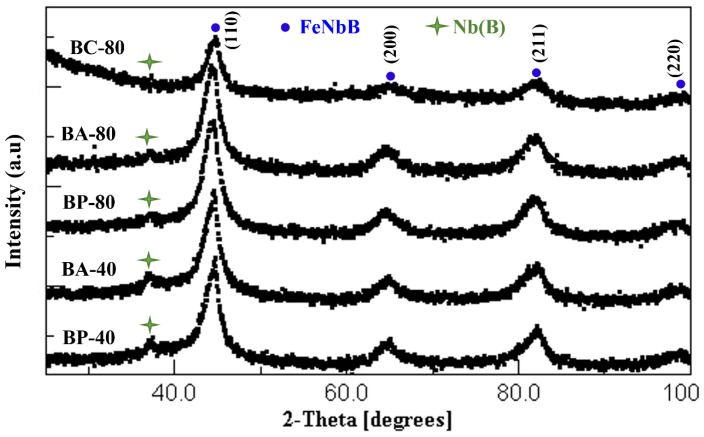
XRD diffractograms for samples of B alloy (Fe_80_Nb_8_B_12_).

Likewise, in terms of checking and analyzing, all samples for both alloys were subjected to XRD at room temperature. Figure 3 and Figure 4 show the diffraction patterns of the obtained powders, agglomerated particles, and compacted specimens of A and B. The Rietveld refinement method was applied to samples with the scientific MAUD software (version 2.8, L. Lutterotti, Trento, Italy).

The Rietveld analysis results show the main crystallographic structure of all the samples examined corresponds to a body-centered cubic (BCC) nanocrystalline structure. The highest intensity peak was detected at an angle 2θ of about 44°. This peak is related to the α-Fe BCC structure (Ref. ICDD code: 01-087-0722). Only the α-Fe rich solid solution phase was found in alloy A milled after 40 and 80 h.

Milling often facilitates the appearance of Fe-B compounds in alloys with Fe and B; however, in Nb-B milling, the Nb(B)-rich phase identified in this study was also reported in the literature [12,13,14,15,16,17,18]. The reaction between B and Nb is attributed to the negative mixing enthalpy which is about −39 kJ/mol. Table 2 and Table 3 display the cell parameter, crystal size, microstrain index (ε), and dislocation densities (*ρ*) for the A (Fe_80_(NiZr)_8_B_12_) and B (Fe_80_Nb_8_B_12_) sample alloys (powder, agglomerated, and compacted), respectively. Relevant refinement parameters are also included. These are the weighted residual (Rwp), expected residual (Rexp), and goodness of fit parameters (GoF).

After 40 h of milling, the powder and agglomerated particles of the A (Fe_80_(NiZr)_8_B_12_) alloy have nearly the same lattice parameter and crystallite size of 2.869 Ǻ and 11 nm, respectively. The agglomerated particles AA-40, on the other hand, have a higher microstrain (ε) of about 0.53% and a higher value of dislocation densities (*ρ*) of about 0.79 × 10^16^ m^−2^. While the analyzed AP-40 has about 0.33% microstrain (ε) and a 0.69 × 10^16^ m^−2^ dislocation density (*ρ*). After 80 h of milling, the lattice parameters of the powder milled and agglomerated particles (AP-80 and AA-80) increased to approximately 2.870 Ǻ and 2.869 Ǻ, respectively. However, an increase in crystallite size was detected to be approximately 14 nm for both samples. Because no significant change in the temperature of the vials after ball milling was found, the increase in particle size after 80 h indicates the powders continue to cold-weld together during ball milling [19,20]. The measured microstrains (ε) were 0.55% and 0.61%, respectively, and their dislocation densities (*ρ*) were 0.47 × 10^16^ m^−2^ and 0.50 × 10^16^ m^−2^, respectively. According to the microstructure analysis of the A (Fe_80_(NiZr)_8_B_12_) alloy, the agglomerated particles have a higher microstrain and dislocation density than the powders milled after the same milling time, despite having nearly the same values of lattice parameter and crystallite size. The compact specimen of the powders milled after 80 h shows the lowest lattice parameter of nearly 2.869 Ǻ and the highest crystallite size of about 26 nm. The dislocation density (*ρ*) and microstrain (ε) were reduced to 0.30% and 0.14 × 10^16^ m^−2^, respectively.

For the samples of the B (Fe_80_Nb_8_B_12_) alloy milled for 40 h, the microstructure results indicate the agglomerated particles have a higher lattice parameter ~2.877 Ǻ and a larger crystallite size (12.15 nm) than the powders milled (2.877 Ǻ, 8 nm, respectively). This behavior is consistent with SEM images, which showed the agglomerated particles obtained after 40 h seemed to have large diameters. Consequently, despite having nearly the same crystallite size, the agglomerated particles have a higher lattice parameter (2.880 Ǻ) than the milled powders (2.876 Ǻ) after 80 h of milling. As with the A alloy, the agglomerated samples obtained after 40 and 80 h of the B alloy show greater microstrain and dislocation density than the milled powders. After 80 h, the compact specimen of the powders milled has a lattice parameter of nearly 2.879 Ǻ and a crystallite size of about 15 nm. The microstrain (ε) and dislocation density (*ρ*) were reduced to 0.44% and 0.41 × 10^16^ m^−2^, respectively. For applications, the powdered mechanically alloyed samples should be compacted to obtain bulk pieces. To preserve the functional properties, it is essential the compaction process does not affect the microstructure. The XRD analysis determined the nanocrystalline structure was retained. Nevertheless, a NiO minor phase ~5.51% was detected on the specimen compacted after milling the A alloy for 80 h (AC-80). Similarly, a minor phase associated to Nb(B)-rich solid solution is found in all B samples.

### 3.3. Thermal Analysis

**Figure 5:**
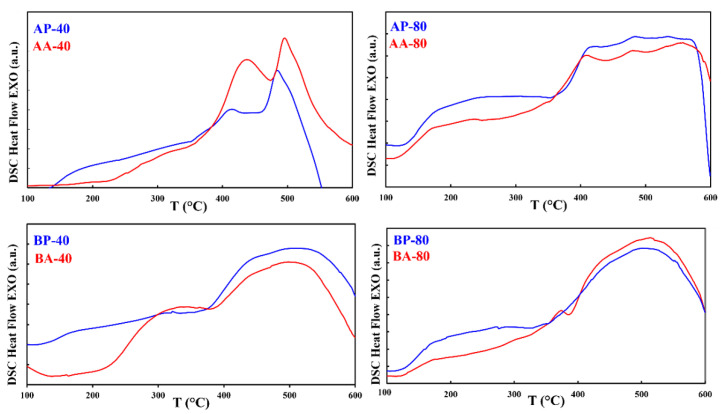
DSC curves collected in pairs for samples (AP-40 and AA-40), (AP-80 and AA-80), (BP-40 and BA-40), and (BP-80 and BA-80) to observe the behavior of powders and agglomerated particles after the same milling duration.

**Figure 6:**
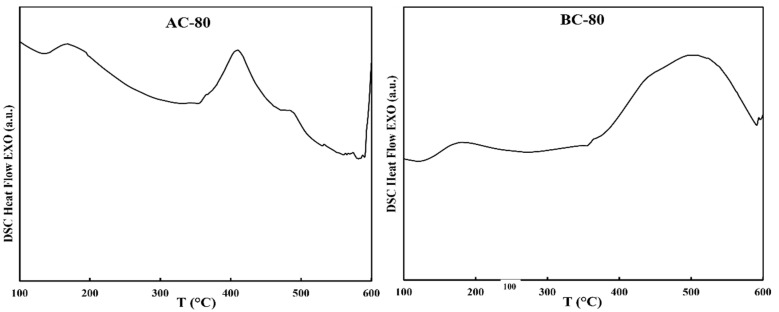
DSC curves of the compacted specimens AC-80 and BC-80.

To check the thermal stability of the nanostructured alloys, DSC analysis was performed, as shown in Figure 5 and Figure 6. The general tendencies of the DSC traces for the powders and agglomerated particles are similar for each milling time. On the one hand, exothermic processes at low temperatures (below 400 °C) in alloy A (AP-40, AA-40, AP-80, and AA-80) were associated to relaxation phenomena generated by a reduction in free volume when mechanically induced tensions were relaxed at micro and nanoscale. Moreover, thermal treatment promotes atomic diffusion, which reduces local inhomogeneity and crystallographic defects. On the other hand, the same relaxation behavior was exhibited in the alloy B (BP-40, BA-40, BP-80, and BA-80) at low temperatures (between 300 and 400 °C). Higher temperature exothermic processes seen in both alloys were attributed to crystallization (crystal growth and/or nucleation). This complicated behavior is characteristic of mechanically alloyed nanocrystalline alloys [21] and is favored by non-homogeneity when Ni or Co are added to Fe-rich alloys. The peak temperature of the powders AP milled after 40 h is lower (T_p_~484 °C) than the temperature of the agglomerated particles AA produced after the same milling time (T_p_~495 °C). After 80 h, the peak temperatures of the powders and agglomerated particules were nearly identical at 483 °C and 482 °C, respectively. For the alloy B, the powders milled and the agglomerated particles obtained after 40 and 80 h did not shown a noticeable difference. For all samples of the B alloy, a wide exothermic process beginning at 400 °C might be caused by early surface crystallization (particle surface) combined with stress [22].

Ultimately, exotermic processes corresponding to relaxation phenomena were found in samples compacted at temperatures below 200 °C. (AC-80 and BC-80). At 400 °C for AC-80 and 500 °C for BC-80, the exotermic peak related with the crystallization process was observed.

Likewise, we analyzed the kinetic of the mean crystallization process. The apparent activation for the main crystallization processes E, was determined using the Kissinger linear approach at heating rates of 5, 10, 20, 30, and 40 K. The Kissinger approach depends on the relation between peak temperature, T_p_, and heating scan rate, β [23].

**Figure 7:**
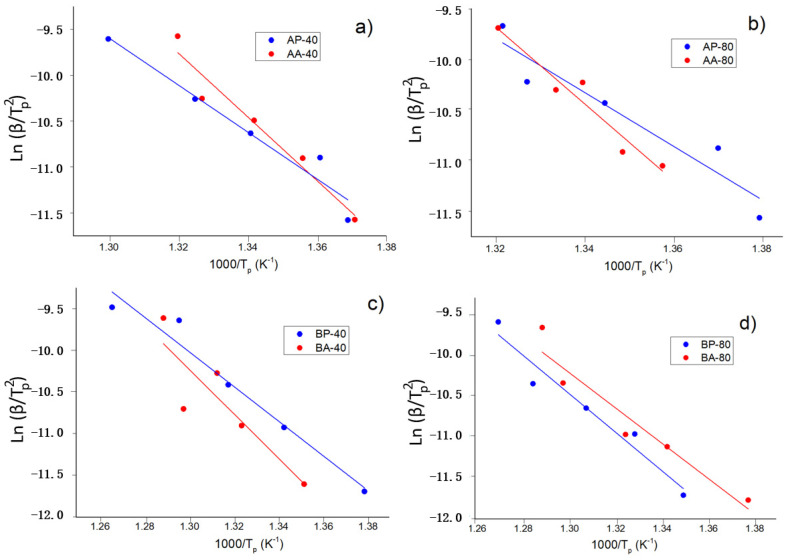
Kissinger linear fitting to determine the activation energy of the crystallization process of the powders and agglomerates (each experimental point corresponds to peak temperatures measured at 5, 10, 20, and 40 K/min). (**a**) Sample A-40h, (**b**) sample A-80h, (**c**) sample B-40h, and (**d**) sample B-80h. Activation energy (and its uncertainity) are given in Table 4.

**Figure 8:**
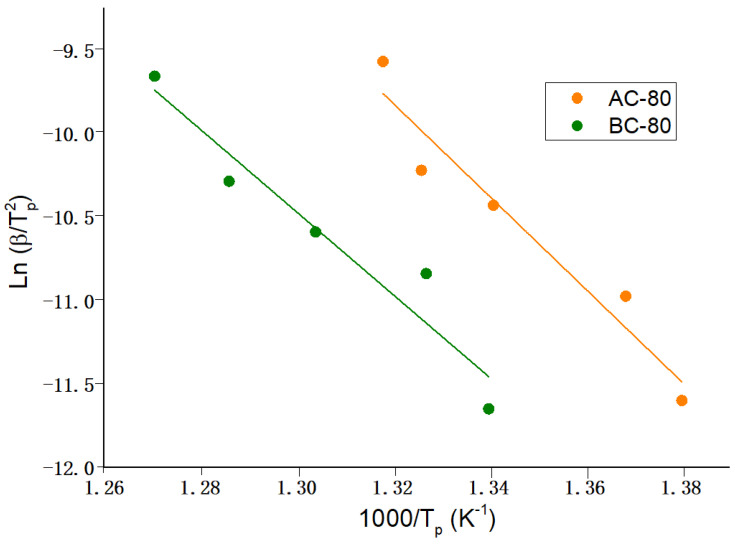
Kissinger linear fitting to determine the activation energy of the crystallization process of the compacted specimens (each experimental point corresponds to peak temperatures measured at 5, 10, 20, and 40 K/min). Activation energy (and its uncertainity) are given in Table 4.

Figure 7 and Figure 8 depict the linear fitting, while Table 4 provides the apparent activation energy. Using the following relation, the apparent activation energy is calculated from the slope of the linear fit where R is the universal gas constant:

(1) E=−(slope·R),

For alloys A and B, the activation energies are higher in agglomerated particles than in powdered particles (at 40 h of milling). A possible explanation is diffusion and homogeneity inside the agglomerated particle stabilize against crystal growth, resulting in a higher activation energy. The same effect is found in alloy A milled for 80 h, whereas the opposite effect is found in alloy B milled for 80 h. According to the Rietveld refinement, the BA-40 and BP-40 powders have approximately the same amount of Nb(B), 3.8% and 3.0%, respectively. The alloy B milled for 80 h exhibited an opposite behavior. The activation energy of the agglomerated particles BA-80 is lower than that of the powders BP-80. One probable reason is the high amount of Nb(B) phase ~5% in the BA-80, which might have affected nucleation and/or crystal formation. The BP-80 presented approximately 1.9% of the Nb(B) phase. Probably, further annealing treatments (followed by XRD analysis) permit arriving at a complete explanation. Finally, this finding also demonstrates the B alloy samples have higher thermal stability than the A alloy samples. This behavior can be attributed to Nb having a greater heat of solution in the Fe matrix than Ni and Zr.

The activation energy of the compacted specimens is similar to the activation energy of the original powders as produced by mechanical alloying.

### 3.4. Magnetic Analysis

**Figure 9:**
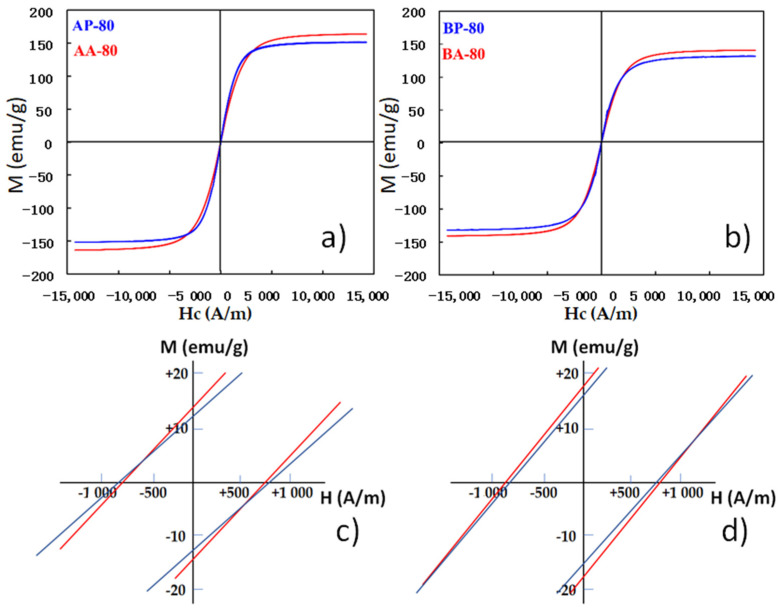
Magnetic hysteresis loops of samples: (**a**) AP-80, AA-80, (**b**) BP-80 and BA-80 and insets of the (0,0 region), (**c**). AP-80, AA-80, (**d**) BP-80 and BA-80.

Likewise, we check the functional soft magnetic response. Magnetic hysteresis cycles at room temperature for alloys A and B are depicted in Figure 9. This type of hysteresis cycle is commonly observable for nanostructured materials with small magnetic domains. This is due to the existence of structural defects inside grains as well as a high density of nanocrystals, which inhibits domain wall movements.

Table 5 summarizes the results of the most relevant magnetic parameters: coercivity, remanent magnetization (Mr), and saturation magnetization (Ms). Magnetic characteristics are affected by sample microstructure parameters, such as crystallite size, particle shape, structural defects, etc. All the examined materials exhibit ferromagnetism in the nanocrystalline state at room temperature and have low coercivity (Hc) close to 770-833 A/m, which is one of the most significant criterions for a soft magnetic material; coercivity values lower than 1000 A/m are associated with soft magnetic materials [24]. The Mr/Ms values are comparable to those obtained in alloys produced by mechanical alloying [25]. 

Regarding the bulk compacted specimens (after 80 h of milling), the coercivity was measured. The process consists of pressing at 600 MPa for 30 min in vacuum. The dimensions of the dies are 10mm in diameter and about 3 mm thick. The analysis of the DSC scans suggests the best annealing temperature to favor the structural relaxation of the samples without inducing the crystal growth is close to 300 °C; annealing was performed at 300 and 600 °C. Higher temperatures are not recommended due to the formation of magnetically undesired intermetallic compounds, such as Fe_3_B [26]. Table 6 shows the coercivity values of the associated average (five measurements in two samples of the same composition) and its statistical error.

The coercivity values of compacted powder milled for 80 h (AC-80) were slightly higher than those of both specimens as milled. We found annealing for 1 h at 300 °C enhanced the coercivity soft behavior (by reducing their values about 5%). It was an expected result because DSC scans show a broad exothermic process linked to structural relaxation at low temperatures. Thus, annealing favors the reduction of dislocation, vacancies, and other defects typical of mechanically alloyed specimens [27]. Likewise, by annealing at 600 °C, the coercivity increases and the compacted material have a soft-hard behavior. This effect is due to the crystallization at temperatures higher than 300 °C (detected as exothermic peaks in DSC scans). The tendency of the results as a function of annealing temperature is the same to those found in specimens with similar composition in previous studies [28].

## 4. Conclusions

In a planetary high-energy ball mill, two nanocrystalline Fe_80_(NiZr)_8_B_12_ (A) and Fe_80_Nb_8_B_12_ (B) alloys were produced. The primary aim of the research was to see whether the powders and agglomerated particles generated during milling had the same microstructure, structural, thermal, and magnetic characteristics.

The structural analysis of the two alloy samples reveals the main phase of all samples was a BCC α-Fe solid solution. A low percentage of Nb(B) phase was identified in the B samples (powders and agglomerated particles). The A alloy’s crystalline size varies between 11 and 14 nm, whereas for B alloys, it ranges between 8 and 12 nm. Agglomerated samples of both alloys had greater microstrain and dislocation density than milled powders.

Additional thermal analysis allowed us to identify the analyzed crystallization process is directly attributable to crystal growth, and thermal stability is higher in agglomerated samples except for the BP-80 that reveals higher stability than the BA-80, which we explained by the higher Nb(B) amount in BA-80.

Magnetic studies at room temperature of all samples milled after 80h revealed soft magnetic behavior with reduced coercivity values in the 770-833 A/m range. Annealing at 300 °C reduces the coercivity of compacted specimens.

## Figures and Tables

**Table 1:** Samples analyzed in the research with the corresponding label. MA signifies mechanical alloying.

Samples	A = Fe_80_(NiZr)_8_B_12_	B = Fe_80_Nb_8_B_12_
MA for 40 h (powder)	AP-40	BP-40
MA for 40 h (agglomerate)	AA-40	BA-40
MA for 80 h (powder)	AP-80	BP-80
MA for 80 h (agglomerate)	AA-80	BA-80
MA for 80 h (compacted)	AC-80	BC-80

**Table 2:** Crystallographic parameters and Rietveld refinement results obtained by Rietveld refinement of the A (Fe_80_(NiZr)_8_B_12_) alloy.

Samples	a (Ǻ)	Crystallite Size (nm)	ε (%)	*ρ* (10^16^ m^−2^)	Rwp (%)	Rexp (%)	GoF
AP-40	2.869(4)	11.98	0.33 (6)	0.69 (6)	16.50	15.257	1.081
AA-40	2.869(3)	11.19	0.53 (1)	0.79 (8)	17.456	16.27	1.072
AP-80	2.870(1)	14.47	0.55 (1)	0.47 (7)	14.920	13.552	1.100
AA-80	2.869(9)	14.05	0.61 (3)	0.50 (6)	16.153	14.396	1.121
AC-80	2.869(1)	26.11	0.30 (1)	0.14 (6)	17.258	13.585	1.270

**Table 3:** Crystallographic parameters and Rietveld refinement results obtained by Rietveld refinement of the B (Fe_80_Nb_8_B_12_) alloy.

Samples	a (Ǻ)	Crystallite Size (nm)	ε (%)	*ρ* (10^16^ m^−2^)	Rwp (%)	Rexp (%)	GoF
BP-40	2.876(1)	8.21	0.34 (3)	1.48 (3)	18.657	14.924	1.250
BA-40	2.876(9)	12.15	0.46 (8)	0.67 (7)	20.490	14.26	1.43
BP-80	2.876(4)	12.02	0.48 (7)	0.69 (2)	16.36	13.17	1.241
BA-80	2.880(9)	11.78	0.51 (1)	0.72 (1)	16.09	13.65	1.178
BC-80	2.879(7)	15.45	0.44 (6)	0.41 (8)	14.89	13.49	1.06

**Table 4:** The activation energy of the main crystallization process in samples A and B.

Samples	Activation Energy/kJ mol^−1^	Samples	Activation Energy/kJ mol^−1^
AP-40	214 (6)	BP-40	173 (13)
AA-40	290 (4)	BA-40	222 (6)
AP-80	221 (18)	BP-80	199 (5)
AA-80	314 (9)	BA-80	182 (3)
AC-80	231 (20)	BC-80	205 (10)

**Table 5:** Relevant parameters determined from the magnetic hysteresis loops of samples AP-80, AA-80, BP-80, and BA-80.

Samples	H_c_ (A/m)	M_r_ (emu/g)	M_s_ (emu/g)	M_r_/M_s_
AP-80	770	13.8	151.0	0.091
AA-80	785	11.5	163.6	0.007
BP-80	812	18.1	131.3	0.138
BA-80	833	17.3	140.8	0.123

**Table 6:** Coercivity, H_c_, of compacted specimens at room temperature (RT) and after annealing (30 min) at 300 or 600 °C.

Sample	H_c_ (RT) /A m^−1^	H_c_ (300 °C) /A m^−1^	H_c_ (600 °C) /A m^−1^
Fe_80_(NiZr)_8_B_12_	827	784	1873
Fe_80_Nb_8_B_12_	845	807	2012

## Data Availability

Data can be requested to the authors.
